# Effect of Biostimulation Using Sewage Sludge, Soybean Meal, and Wheat Straw on Oil Degradation and Bacterial Community Composition in a Contaminated Desert Soil

**DOI:** 10.3389/fmicb.2016.00240

**Published:** 2016-03-04

**Authors:** Sumaiya Al-Kindi, Raeid M. M. Abed

**Affiliations:** Biology Department, College of Science, Sultan Qaboos UniversityMuscat, Oman

**Keywords:** desert soil, oil, illumina, bioremediation, sewage sludge, soybean meal, wheat straw

## Abstract

Waste materials have a strong potential in the bioremediation of oil-contaminated sites, because of their richness in nutrients and their economical feasibility. We used sewage sludge, soybean meal, and wheat straw to biostimulate oil degradation in a heavily contaminated desert soil. While oil degradation was assessed by following the produced CO_2_ and by using gas chromatography–mass spectrometry (GC–MS), shifts in bacterial community composition were monitored using illumina MiSeq. The addition of sewage sludge and wheat straw to the desert soil stimulated the respiration activities to reach 3.2–3.4 times higher than in the untreated soil, whereas the addition of soybean meal resulted in an insignificant change in the produced CO_2_, given the high respiration activities of the soybean meal alone. GC–MS analysis revealed that the addition of sewage sludge and wheat straw resulted in 1.7–1.8 fold increase in the degraded C_14_ to C_30_ alkanes, compared to only 1.3 fold increase in the case of soybean meal addition. The degradation of ≥90% of the C_14_ to C_30_ alkanes was measured in the soils treated with sewage sludge and wheat straw. MiSeq sequencing revealed that the majority (76.5–86.4% of total sequences) of acquired sequences from the untreated soil belonged to Alphaproteobacteria, Gammaproteobacteria, and Firmicutes. Multivariate analysis of operational taxonomic units placed the bacterial communities of the soils after the treatments in separate clusters (ANOSIM *R * = 0.66, *P* = 0.0001). The most remarkable shift in bacterial communities was in the wheat straw treatment, where 95–98% of the total sequences were affiliated to Bacilli. We conclude that sewage sludge and wheat straw are useful biostimulating agents for the cleanup of oil-contaminated desert soils.

## Introduction

Oil contamination results in a dramatic increase in carbon sources in affected soils and a depletion of important nutrients such as nitrogen and phosphorus (Boufadel et al., 1999; Hazen, 2010). This imbalance in carbon–nitrogen ratio and the nitrogen deficiency hamper the biodegradation process (Chorom et al., 2010). Waste materials, such as sewage sludge and soybean meal (termed hereafter as SG and SB, respectively), are potential stimulating agents for bioremediation as they are economically feasible and rich in nutrients such as phosphorus, nitrogen and carbon (Cui et al., 2008; Agamuthu et al., 2013). SG has been used as an organic fertilizer, a soil ventilator and a bioaugmentation agent in the bioremediation of oil- and metal-contaminated soils (Gallego et al., 2001; Hamdi et al., 2006; Chorom and Hosseini, 2011; Park et al., 2011). The addition of SG reduced up to 45% of polyaromatic hydrocarbons (PAHs) and 43–98% of total petroleum hydrocarbons (TPHs) in different contaminated soils (Hur and Park, 2003; Chorom et al., 2010; Ros et al., 2010; Zhang et al., 2012). SB has also been used as a source of organic nitrogen in the bioremediation of several oil-contaminated soils (Diab, 2013). The addition of SB has been shown to enhance biosurfactant production and to increase hydrocarbons bioavailability (Diab and Sandouka, 2012).

Besides nutrients, oxygen is another limiting factor that influences the efficiency of bioremediation processes especially that the first step in the breakdown of hydrocarbons under aerobic conditions relies on oxygen-dependent enzymes (Gallego et al., 2001). Different aeration methods, such as titling, forced aeration and addition of bulking agents, were employed to accelerate bioremediation (Rhykerd et al., 1999; Odokuma and Dickson, 2003; Marin et al., 2006; Hu et al., 2012). The function of bulking agents is to increase soil porosity, increase oxygen diffusion, lower soil’s bulk density and provides a valuable carbon and energy source for microorganisms (Rhykerd et al., 1999; Lang et al., 2000; Huang et al., 2006). Wheat straw (termed hereafter as WS), wood chips, post-peelings, peanut powder were successfully used as aerating agents to biostimulate the growth and activity of microorganisms in PAH-contaminated soils (Llado et al., 2015).

The bioremediation of desert soils is challenging, mainly because of the harsh environmental conditions. The diversity of microorganisms in oil-contaminated arid deserts and their response to different bioremediation treatments have been relatively much less studied than marine sediments. In the Arabian Peninsula, oil spills are very common in deserts, nevertheless little research has been performed to study their environmental impacts and remediation (Radwan, 2008, 2009). Here, we investigate the effect of the biostimulators SG, SB, and WS on respiration activities, hydrocarbons degradation and bacterial community changes in oil-contaminated soils from the desert of Oman. To the best of our knowledge, the response of bacterial activity and diversity in oil-contaminated desert soils to three biostimulating agents has been rarely compared in a single study and using next generation high throughput sequencing (NGS). Recently, NGS has become a robust and a straightforward technology with the ability to generate large sequence databases in a massively parallel fashion (Shokralla et al., 2012). This technique has yielded comprehensive information on the structure of microbial communities and their shifts in contaminated sites (Singleton et al., 2013; Lamendekka et al., 2014; Sun et al., 2015).

## Materials and Methods

### Collection of Soils and Organic Wastes

Oil-contaminated soils were collected on May, 2013 from a dumping area in the deserts of Marmul, Southwest of Muscat, Sultanate of Oman (lat. 18° 10′ 01.3″N; long. 55° 14′ 32.9″E). The area is located close to an oil exploration field and used to collect the oil-contaminated soils, so crude oil was the sole contaminant in the region. Approximately 500 g of the top 1 cm of the soils were collected in triplicates from three locations that were 50–100 m apart. TPH in the soils were gravimetrically measured after extraction of 5 g of soil using 15 ml of dichloromethane (DCM, Sigma-Aldrich, Germany) for 2–3 times. The extracts were filtered by non-absorbent cotton and then subjected to evaporation by rotary evaporator to quantify TPH (Weisman, 1998). The pH and electrical conductivity (EC) were measured using the filtrates of 10 g soil mixed with 50 ml of deionized water, using calibrated YSI instruments. Anions were extracted and evaluated using ion chromatography (IC, Metrohm AG, Herisau, Switzerland). The percent of sand, silt and clay in the soil was determined by a standard hydrometer method (Klute, 1986), and this percent was used to categorize the soil texture from a soil triangle (Brady, 1984).

SB and WS were collected from the Gulf Mushroom Products Company (S.A.O.G) in Barka, Oman. The average moisture and nitrogen contents of the SB were 10 and 8%, whereas these values were 12 and 0.5% for the WS, respectively. SG was collected from an aeration tank in the wastewater treatment plant at Sultan Qaboos University (SQU).

### Biostimulation Experiment in Glass Bottles

Biostimulation experiments were conducted in closed glass bottles (termed hereafter as bottle experiment) in order to follow respiration activities of untreated and treated soils through measuring the amount of produced CO_2_ and to measure oil degradation at the end of the treatments using gas chromatography–mass spectrometry (GC–MS). One gram of oil-contaminated soil was placed in 165 ml serum glass bottles. To this, 50 mg of each of the biostimulating agents (i.e., SG, SB, and WS) were added. Five ml of sterilized water were added to each vial. Two controls were maintained; one with contaminated soil but without any treatment (untreated soil) and the other with only the biostimulating agent without soil. All treatments and controls were maintained in triplicates. All vials were sealed with a butyl rubber stopper and an aluminum crimp cap to ensure no gas leakage and were incubated for 110 days in the oven at 30°C without shaking. The produced CO_2_ was measured by withdrawing 250 μl from the headspace of the bottle using a gas-tight glass syringe and injecting manually into gas chromatography (GC, Agilent model 6890N). The GC was equipped with a thermal conductivity detector and a 30 m × 250 μm capillary column (HP-PLOT Q). Helium was used as a carrier gas at a flow rate of 4 ml min^-1^ and the injector and detector temperatures were maintained at 200 and 210°C, respectively. The oven temperature was programmed from 50 to 80°C (final hold time 3 min) at a rate of 20°C min^-1^. Carbon dioxide (CO_2_) evolution data were statistically analyzed by one-way ANOVA using the SPSS software (10th edition, Chicago, IL, USA). *P*-values were adjusted using the sequential Bonferroni (Quinn and Keough, 2002) and Tukey’s test was used to determine differences between individual means. The degradation of the oil in the bottle experiment was assessed using GC–MS analysis of the alkanes (see below). The ratios of degraded alkanes to the amount of produced CO_2_ in the SG and WS treated soils were calculated, after subtracting the CO_2_ produced from soil-free SG and WS.

### Biostimulation Experiment in Microcosms

Since the laboratory experiment was performed in closed bottles to enable the measurement of CO_2_ gas in the headspace, oxygen could become limited and this could slow down biodegradation processes. Therefore, another experiment was performed in open-air glass aquaria (termed hereafter as microcosm experiment) in order to follow the degradation using GC–MS and to monitor shifts in the bacterial community structure. Here, 100 g of soil were placed in glass aquaria (15 cm diameter). Five grams of each of the biostimulating agents (i.e., SG, SB, and WS) were added individually to each aquarium. Untreated soils were kept as controls. All treatments and controls were maintained in triplicates. The biostimulating agents were mixed with the soil, and the mixture was tilled twice a week using a sterile spatula. The mixture was always kept wet by adding 10 ml of sterile water every 2 days. All incubations were kept for 64 days at ca. 30 ± 3°C in a greenhouse. Samples (i.e., 1 g soil each) were collected for GC–MS analysis and MiSeq illumina (see below).

Oil biodegradation (mainly C_11_ to C_30_ alkanes) from the microcosm experiments (and from the bottle experiments) was followed using GC–MS analysis of the soils at the end of the experiment. One gram of each soil was extracted three times in 5 ml dichloromethane (DCM, Sigma-Aldrich, Germany) and then sonicated for 25 min at 10°C. The extracted supernatant was mixed with sodium sulfate and was filtered with non-absorbent cotton to remove solid particles. The filtrate was then evaporated using a rotary evaporator. The dry extract was re-dissolved into DCM and passed through silica gel prior to injection into GC–MS (Perkin Elmer Clarus 600GC/MS). The Perkin Elmer Clarus 600C MS was coupled with Rtx^®^-5MS capillary column (30 m × 0.25 mm I.D. × 0.25 μm film thickness; maximum temperature, 350°C). Ultra-high purity helium was used as a carrier gas at a constant flow of 1.0 ml/min. The ionizing energy was 70 eV. Electron multiplier (EM) voltage was obtained from autotune. The injection, transfer line and ion source temperatures were 290, 280, and 280°C, respectively. The oven temperature program was held at 80°C for 5 min and then accelerated at a rate of 10°C/min to 280°C (hold for 30 min). The volume of injected sample was 1 μl with a split ratio of 10:1. The standard mix solution (C_7_ to C_30_) of concentrations 10, 20, 30, 40, and 50 ppm were used for confirmation and quantification purposes. A calibration curve conformed by the standard mixtures was established and quantification of the analyzed compounds was performed in the linear range of the calibration curve. The alkanes were identified based on GC retention times of the standard, injected and analyzed under the same conditions as samples and by comparing the spectra obtained with mass spectrum libraries (NIST 2011 v.2.3 and Wiley, 9th edition).

### Bacterial Community Analysis using Illumina

DNA was extracted from the soils at the end of the microcosm experiment using skim milk protocol (Volossiouk et al., 1995). Purified DNA extracts were then submitted to Molecular Research MR DNA laboratory (www.mrdnalab.com, Shallowater, TX, USA) for illumina MiSeq sequencing of the bacterial 16S rRNA genes using the primers 341F (5′-CCTACGGGNGGCWGCAG-3′) and 805R (5′-GACTACHVGGGTATCTAATCC-3′) with barcode on the forward primer (Klindworth et al., 2013). After amplification, PCR products were checked in 2% agarose gel to determine the success of amplification and the relative intensity of bands. Multiple samples were pooled together in equal proportions based on their molecular weight and DNA concentrations. Pooled samples were purified using calibrated AMPure XP beads. Then, the pooled and purified PCR products were used to prepare a DNA library by following illumina TruSeq DNA library preparation protocol. Sequence analysis was carried out using the Mothur MiSeq SOP pipeline (Schloss et al., 2009). Briefly, barcodes were removed and sequences <200 bp and sequences with ambiguous base calls were eliminated. Sequences were denoised, operational taxonomic units (OTUs) generated and chimeras removed. OTUs were defined by clustering at 3% divergence (97% similarity). Final OTUs were taxonomically classified using BLASTn against a curated GreenGenes database (De Santis et al., 2006). Rarefaction curves and diversity indices (OTU richness, Chao-1, and ACE) were calculated using the Mothur software. A multivariate analysis of all samples was performed to examine for significant changes in soil communities after biostimulation treatments using non-metric multidimensional scaling (NMDS) based on Bray–Curtis dissimilarities as described in Ramette and Tiedje (2007). Analysis of similarities (ANOSIMs) was carried out to test for significant differences in bacterial communities. ANOSIM produces a sample statistic (*R*), which represents the degree of separation between test groups (Clarke, 1993).

## Results

### Physicochemical Characteristics of the Desert Soil

The TPH content in the studied soil was 41.71 mg g^-1^ soil (**Table 1**). The soil had a neutral pH. Soil texture was classified as silt loam as it contains more silt than sand and clay. The concentrations of nitrate and phosphate were 0.04 and 0.16 mg g^-1^ soil, respectively (**Table 1**). Chloride, bromide, fluoride, and sulfate were measured at detectable amounts (**Table 1**). This desert soil contained no or very little amounts of biogenic matter.

**Table 1:** Physicochemical properties of the studied oil-contaminated desert soil.

		Unit	Desert soil
**Parameters**		
TPH		mg g^-1^	41.71
pH			7.50
EC		mS g^-1^	1.52
Fluoride		mg g^-1^	0.03
Chloride		mg g^-1^	32.52
Bromide		mg g^-1^	0.15
Nitrate		mg g^-1^	0.04
Phosphate		mg g^-1^	0.16
Sulfate		mg g^-1^	31.83
**Soil texture**			
	Sand	%	27.00
	Clay	%	23.00
	Silt	%	50.00
	Type		Silt loam


*TPH, Total petroleum hydrocarbons; EC, electrical conductivity at 20°C.*

### Changes in Respiration Activities

**FIGURE 1:**
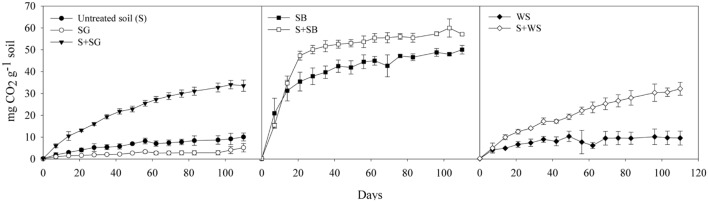
**The cumulative amount of CO_2_ (in mg g^-1^ soil) produced from the untreated and the biostimulated soils.** Note that the produced CO_2_ from the soybean alone (SB) was much higher than from the sewage sludge (SG) alone and the wheat straw (WS) alone. Error bars represent ± standard deviation (*n* = 3).

The produced CO_2_ in the untreated desert soil without any amendment reached 10.1 ± 1.8 mg CO_2_ g^-1^ soil after 110 days of incubation (**Figure 1**; **Table 2**). While the SG and the WS alone produced CO_2_ amounts lower than 10 mg CO_2_ g^-1^ soil, the SB alone yielded a total amount of 50.0 ± 1.9 mg CO_2_ g^-1^ soil (**Figure 1**). The addition of SG and WS to the desert soil significantly (*P* < 0.001) stimulated the respiration activities to reach the values 33.6 ± 2.5 and 32.1 ± 2.9 mg CO_2_ g^-1^ treated soil at the end of the incubation period, respectively (**Table 2**; **Figure 1**). In the case of SB, the produced CO_2_ of the treated soil displayed the highest value of 57.1 ± 0.5 mg CO_2_ g^-1^ soil, however, this value accounts for an insignificant (*P* > 0.05) increase in CO_2_ from the untreated soil, given the high respiration activities of the SB alone.

**Table 2:** The total produced CO_2_ after 110 days of incubation (without any subtraction) and the amount of degraded alkanes (C_14_ to C_30_) in the bottle and the microcosm experiments (in mg g^-1^ soil and in % of initial alkane concentrations in the control). All values represent mean ± standard deviation.

Type of treatment	Total evolved CO_2_ after 110 days (mg-CO_2_ g^-^1 soil)	Alkane (Ci_4_ to C_30_) degradation as measured by GC–MS			
		
		Bottle experiment (110 days)		Mcrocosom experiment (64 days)	
			
		(mg g^-1^)	(%)	(mg g^-1^)	(%)
Untreated soil (S)	10.1 ± 1.8	10.9 ± 0.1	55 ± 0.5	9.7 ± 0.6	50 ± 3.0
S+SG	33.6 ± 2.5	19.0 ± 0.7	95 ± 3.5	18.3 ± 0.7	92 ± 3.5
S+SB	57.1 ± 0.5	14.3 ± 0.4	72 ± 2.0	12.4 ± 0.5	63 ± 2.5
S+WS	32.1 ± 2.9	18.3 ± 0.6	92 ± 3.0	17.9 ± 0.1	90 ± 0.5


*SG, Sludge; SB, soybean meal; WS, wheat straw.*

### Oil Degradation as Revealed by GC–MS

**FIGURE 2:**
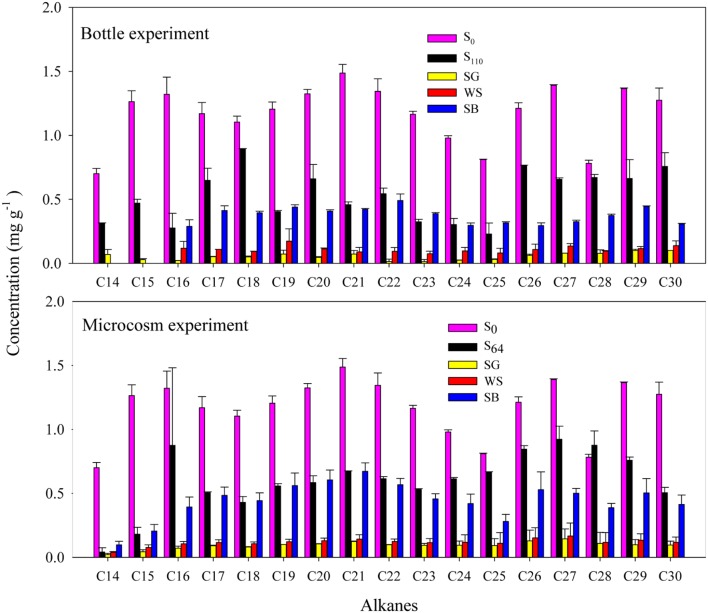
**Concentration of individual alkanes (C_14_ to C_30_) before and after biostimulation with sewage sludge (SG), soybean meal (SB), and wheat straw (WS) in the bottle and the microcosm experiments.** Error bars represent ± standard deviation (*n* = 3).

**FIGURE A1:**
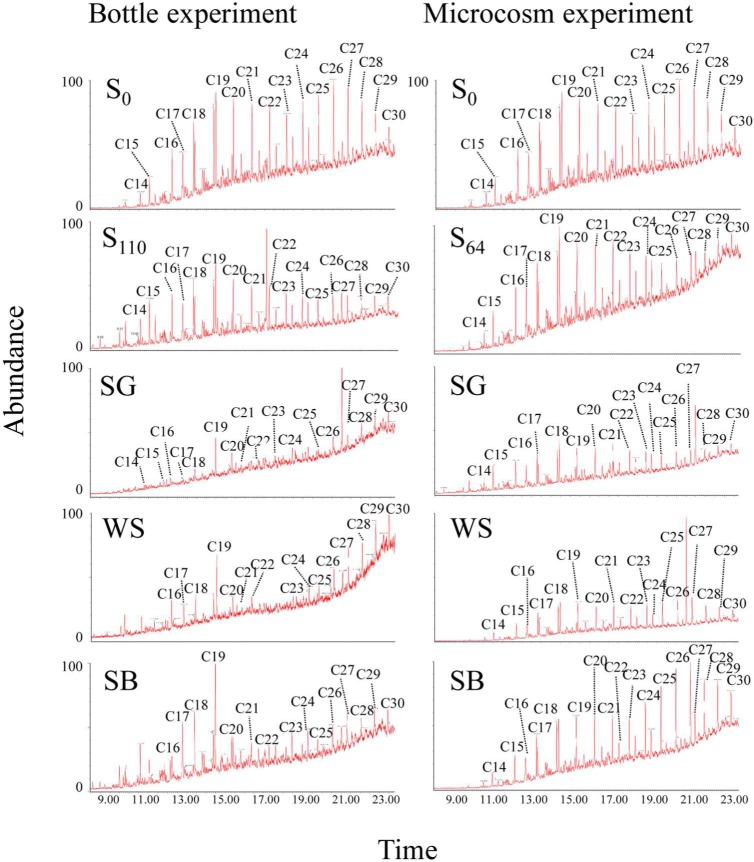
**Gas chromatography/mass spectrometry (GC/MS) chromatograms showing the concentrations of crude oil fractions before and after biostimulation with sewage sludge (SG), wheat straw (WS), and soybean meal (SB) in the bottle and in the microcosm experiments.** S_0_ denotes the original soil before incubation and S_110_ and S_64_ denote the same soil at the end of incubation without any treatment in the bottle and in the microcosm experiment, respectively.

In the bottle experiment, GC–MS revealed that around 10.9 ± 0.1 mg of the C_14_ to C_30_ alkanes g^-1^ soil were degraded in the untreated soil after 110 days of incubation (**Table 2**; **Figure 2**). The lighter fraction of alkanes (<C_14_) completely disappeared from the GC chromatograms (**Appendix Figure A1**). The addition of SG and WS resulted in the degradation of 18.3–19.0 mg of the alkanes g^-1^ soil at the end of the experiment. These values correspond to the degradation of >90% of the total alkanes at a degradation rate of 0.28–0.29 mg of the alkanes g^-1^ soil day^-1^. The addition of SB resulted in the degradation of 14.3 ± 0.4 mg g^-1^ of the alkanes in 110 days (**Table 2**; **Figure 1**). The degradation of C_14_ to C_30_ alkanes in the microcosm experiment exhibited a similar pattern to that of the bottle experiment. The maximum amount of alkane degradation was 18.3 ± 0.7 mg g^-1^ soil after 64 days of treatment with SG (**Table 2**). This amount corresponds to a degradation rate of 0.29 mg of the alkanes g^-1^ soil day^-1^.

### Bacterial Community Changes

**FIGURE 3:**
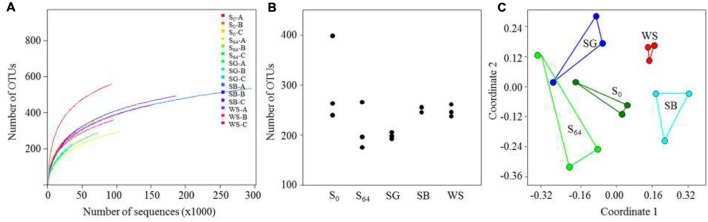
**(A)** Calculated rarefaction curves of observed OTU (sequences that have 97% similarity are defined as one OTU) richness in the soils; **(B)** number of OTUs calculated for the triplicates of each soil sample, using the same number of sequences for all soils; **(C)** non-metric multidimensional scaling (NMDS) ordination (based on a Bray–Curtis distance matrix) of the triplicates of each treatment.

The total number of generated 16S rRNA gene sequences by illumina MiSeq was 1,395,417 reads. The lowest number of sequences per sample was 27585 and the highest was 295156 (**Table 3**). Rarefaction curves showed that no samples, regardless of the number of sequences, reached a maximum yield of OTUs (**Figure 3A**). The number of OTUs, calculated using subsets with the same number of sequences for all samples, was in the range of 240–400 OTUs in the untreated soil (**Table 3**; **Figure 3A**). The OTU richness decreased in the untreated soil at the end of the experiment after 64 days of incubation to reach 176–266 OTUs (**Figure 3B**). In the treated soils, the number of OTUs was lowest in the case of the SG with an average of 199 ± 6, whereas the average reached 252 ± 6 and 249 ± 12 in the case of the SB and the WS, respectively (**Figure 3B**). When variations in bacterial community composition were visualized in a two-dimensional space using multivariate analyses of OTUs (**Figure 3C**), the bacterial communities of the triplicates of each treatment were placed in separate clusters (**Figure 3C**, ANOSIM *R* = 0.66, *P* = 0.0001).

**Table 3:** MiSeq sequencing and bacterial diversity estimators for the untreated and the biostimulated soils (S_0_: original soil; S_64_ untreated soil after 64 days of incubation; SG: soil treated with sewage sludge; SB: soil treated with soybean meal and WS: soil treated with wheat straw).

Sample	Replicate	Total No. of sequences	No. of OTUs^∗^	Chao-1	ACE
S_0_	A	92635	399	540.2	533.1
	B	43103	263	384.3	411.7
	C	73774	240	385.6	397.2
S_64_	A	104773	176	288.8	308.5
	B	108467	266	407.1	414.0
	C	73415	197	315.0	331.2
SG	A	35845	206	307.2	328.0
	B	33379	198	323.1	320.4
	C	29766	193	317.7	344.1
SB	A	295156	246	374.4	391.6
	B	27585	256	451.0	457.7
	C	186157	255	413.4	427.7
WS	A	44936	262	422.7	447.3
	B	150844	246	396.1	384.1
	C	95582	238	354.5	352.1


*^∗^Operational taxonomic unit at 3% sequence dissimilarity based on equal subsets of sequences for all samples, Chao-1 is based on rare OTUs in a given sample and ACE is abundance-based coverage.*

**FIGURE 4:**
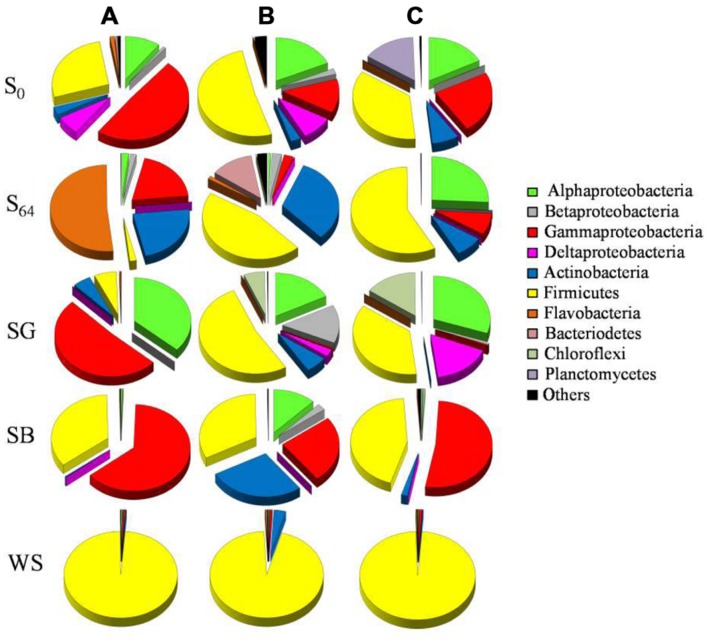
**Pie charts depicting the relative abundance (% of total sequences) of bacterial groups in the original soil (S_0_) and in the soils at the end of the microcosm experiment (S_64_: untreated soil after 64 days of incubation; SG: sewage sludge treatment; SB: soybean meal treatment; WS: wheat straw treatment).** The triplicates of each soil are denoted as **(A–C)**.

MiSeq data showed noticeable changes in bacterial community composition after 64 days of incubation, both in the treated and untreated soils. At the beginning of the experiment, the original soil was dominated by sequences related to the bacterial groups Alphaproteobacteria, Gammaproteobacteria, and Firmicutes (76.5–86.4% of total sequences; **Figure 4**). Sequences belonging to Planctomycetes constituted 15% of the total number of sequences in one of the replica, but <2% in the other two (**Figure 4**). After 64 days of incubation, the relative abundance of Alphaproteobacteria exhibited a decrease only in two replicates to reach 0.6–1.9% of total sequences (**Figure 4**). The bacterial community also showed a decrease in the relative abundance of Gammaproteobacteria from 13–48 to 3–20% of total sequences in all replicates (**Figure 4**). On the other hand, the relative abundance of Firmicutes increased to 46.6–57.8% of total sequences at least in two replicate samples, whereas the third showed an increase in the dominance of Flavobacteria (51.6% of total sequences in S_64_-A, **Figure 4**). Additionally, sequences belonging to the phylum Actinobacteria increased in abundance in all samples (≤32% of total sequences). There was not much change in the relative abundance of the bacterial phyla Betaproteobacteria, Bacteriodetes, and Chloroflexi (**Figure 4**).

**FIGURE 5:**
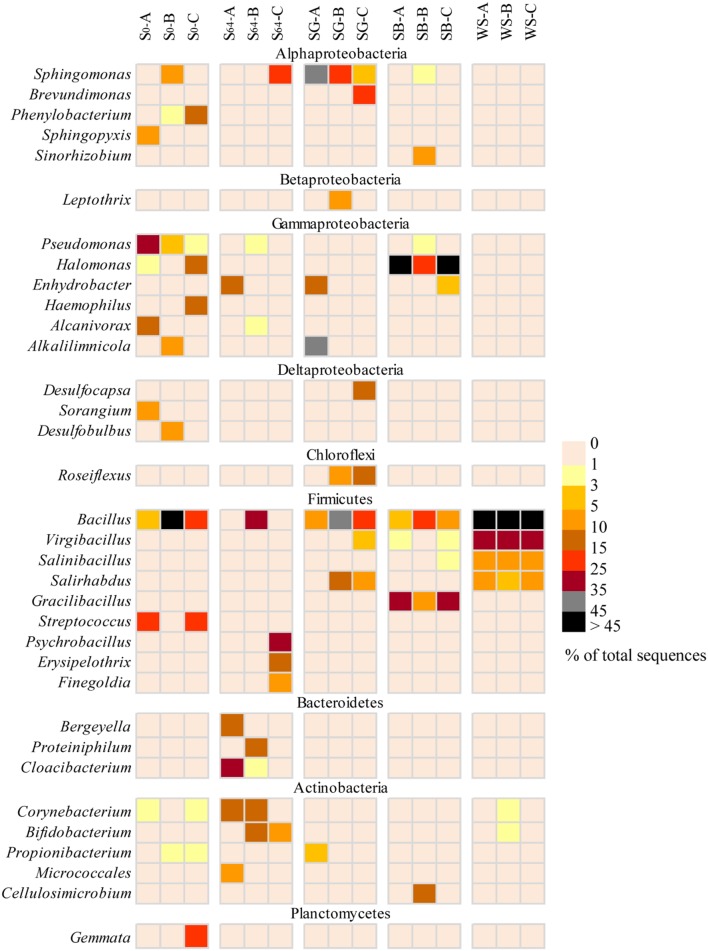
**Heatmap representing a comparison of the relative abundance (% of total sequences) of the major bacterial genera in the most dominant bacterial groups in the different soils.** The triplicates of each soil are denoted as A–C.

At the genera level, the majority of sequences of Alphaproteobacteria in the untreated soil, at the beginning of the experiment, belonged to the genera *Sphingopyxis*, *Phenylobacterium*, and *Defluviicoccus* (**Figure 5**). However, after 64 days of incubation, only sequences related to *Sphingopyxis* persisted in one of the replicas (i.e., S_64_-C). Gammaproteobacteria was dominated by *Pseudomonas*, *Halomonas*, *Haemophilus*, and *Alcanivorx* but after incubation, all these genera disappeared and only in one replica, sequences belonging to the genus *Enhydrobacter* appeared (**Figure 5**). Sequences related to *Bacillus* and *Streptococcus* constituted 85–98% of total sequences of Firmicutes in the original soil. After incubation, *Bacillus* was still detectable, in addition to two new genera *Psychrobacillus* and *Erysipelothrix.* While the phylum Bacteroidetes was dominated by sequences related to *Bergeyella, Proteiniphilum* and *Cloacibacterium*, Actinobacteria was dominated by sequences affiliated to *Corynebacterium*, *Bifidobacterium*, and *Micrococcales* (**Figure 5**).

The addition of SG to the oil-contaminated desert soils induced variable changes in the triplicate samples. While Alphaproteobacteria increased in abundance in all replicates, Gammaproteobacteria increased only in SG-A (51% of total sequences), Betaproteobacteria increased only in SG-B (14%) and Deltaproteobacteria increased only in SG-C (17%). The majority (>98%) of Aphaproteobacteria belonged to the bacterial genera *Sphingomonas* and *Brevundimonas* (**Figure 5**). Sequences of Gammaproteobacteria were related to the genera *Alkalilimnicola* and *Enhydrobacter* whereas sequences belonging to Betaproteobacteria and Deltaproteobacteria were dominated by the genera of *Leptothrix* and *Desulfocapsa*, respectively (**Figure 5**). Actinobacteria was detectable in all replicates, but at a lower abundance (i.e., 0.2–6% of total sequences) than the untreated soil. In contrast, the relative abundance of Chloroflexi increased at least in two replicates to reach ≤15% of the total sequences and the only detectable genus was *Roseiflexus* (**Figure 5**). Most of the Firmicutes sequences were affiliated to *Bacillus, Salirhabdus*, and *Virgibacillus* and were mainly dominating the samples SG-B and SG-C (**Figure 5**).

In the case of the addition of SB, Gammaproteobacteria and Firmicutes constituted between 58 and 99% of total sequences in all soil samples (**Figure 4**). While 91–99% of the gammaproteobacterial sequences were affiliated to the genus *Halomonas*, 89–90% of the Firmicutes sequences belonged to *Bacillus* and *Gracilibacillus* (**Figure 5**). In one replica, Actinobacteria and Alphaproteobacteria made up ca. 12 and 27% of total sequences, respectively whereas in the other two replicas, both bacterial groups made up ≤2.5% (**Figure 4**). These sequences were mainly affiliated to the genera *Sphingomonas*, *Sinorhizobium*, and *Cellulosimicrobium* (**Figure 5**). The remaining bacterial groups remained at a relative abundance of ≤2% of total sequences each in all samples.

The addition of WS to the contaminated soils induced the most remarkable changes in the bacterial community composition. The whole community shifted in the favor of the Firmicutes group, which made up 95–98% of total sequences in all samples (**Figure 4**). The dominating genera in this group were *Bacillus, Virgibacillus, Salinibacillus*, and *Salirhabdus* (**Figure 5**).

## Discussion

Our data demonstrated a greater effect of SG and WS than SB in the stimulation of respiration activities and oil degradation rates in the oil-contaminated desert soil. The addition of these agents has been shown to enrich soils with nutrients, such as phosphorous and nitrogen, whose limitation is known to slow down biodegradation processes (Cui et al., 2008; Chorom et al., 2010; Agamuthu et al., 2013). Although the addition of SB increased oil degradation rates, as revealed by GC–MS analysis, its effect on respiration activities was insignificant. This could be attributed to the active microbial community in the used SB, as suggested by the high amount of produced CO_2_ by the SB alone (50.0 ± 1.9 mg CO_2_ g^-1^ soil). These exogenous bacteria could compete for the available organics and nutrients with the indigenous bacteria in the contaminated soil. Furthermore, it is not clear whether the measured CO_2_, which represents net production, originated from the mineralization of oil components or from other organics in the SB. We speculate that a fraction of the SB and the soil bacterial communities switched between feeding on the SB organics and feeding on hydrocarbons, resulting in the same net production of CO_2_. Although, the addition of SB resulted in an increase of only 30% in alkane degradation in our experiments, previous studies showed an increase in the degradation of motor oil and phenol to reach 60–90% upon the addition of SB and soybean seed hull (Flock, 1998; Diab, 2013). SB was even used in the bioremediation of palm oil mill effluent (Ibegbulam-Njoku and Achi, 2014).

The use of CO_2_ evolution as a measure of oil mineralization has been successfully used in several reports (Boufadel et al., 1999; Kim et al., 2005; Morais and Tauk-Tornisielo, 2009; Abed et al., 2014; Personna et al., 2014). However, in our experiments, we could not reliably calculate oil mineralization from the produced CO_2_, since the added biostimulating agents could act as an additional carbon source to oil. In spite of that, there was a good correlation between the total amounts of degraded alkanes, measured using GC–MS, and the amounts of cumulative produced CO_2_ (**Table 2**) in the untreated and treated soils in the bottle experiment (*r* = 0.75, *P* ≤ 0.01). Such positive correlation, where the produced CO_2_ increases with increasing degradation of hydrocarbons, has been previously observed (Namkoong et al., 2002). The ratios of degraded alkanes to the amount of produced CO_2_ in the SG and WS treated soils, after subtracting the produced CO_2_ from soil-free SG and WS, were 0.66 and 0.81, respectively. Previous studies have reported values between 0.8 and 1.4 (Hwang et al., 2006). Our values were lower than those reported previously, mainly because our calculation was based on the amounts of degraded alkanes, which is a fraction of oil, and not on the total TPH. Moreover, it is known that a fraction of the carbon could be assimilated into biomass (Linton and Stephenson, 1978). Using GC–MS, the degradation of alkanes was slightly higher (*P* < 0.05) in the bottle experiment than in the microcosm experiment. However, when the incubation period is taken into account (i.e., 110 vs. 64 days), the degradation rates were still higher in the microcosm experiment (i.e., 0.172 and 0.166 vs. 0.29 and 0.28 mg of alkanes g^-1^ soil day^-1^, for SG and WS amended soils, respectively). This is due to the clear differences in the incubation conditions between the bottle and the microcosm experiments, including water content, aeration, mixing, and scale. The continuous supply of oxygen in the microcosm experiment due to soil tilting apparently favored oil degradation.

Previous reports have used SG for bioremediation of oil-contaminated soils because of its richness in nutrients and the existence of high diversity and density of microorganisms therein (Chang et al., 2003; Gestel et al., 2003; Ros et al., 2010; Ambrazaitiene et al., 2013). However, the low respiration activities of SG suggests a low activity of its microbial community. The remarkable increase in the produced CO_2_ after the addition of SG to the contaminated soil indicates that SG was most likely a good source of nutrients. Previous reports showed that the addition of SG to diesel- and oil-contaminated soils resulted in a total degradation rate of 46–98% of TPHs (Namkoong et al., 2002; Hur and Park, 2003; Ros et al., 2010; Chorom and Hosseini, 2011). Although, the use of 5% (w/w) SG in our experiments was effective, the use of as high as 10 and as low as 1% was also shown to stimulate oil degradation rates (Hur and Park, 2003; Ros et al., 2010; Chorom and Hosseini, 2011). On the contrary, the excessive use of SG retarded the biodegradation of TPH in other cases (Namkoong et al., 2002).

The bioventing agent, WS, stimulated respiration activities and oil mineralization rates in the oil-contaminated desert soils. Since the studied desert soil had a silt loam texture with 50% silt, it is plausible that the addition of WS resulted in an increase in the soil porosity and oxygen availability to the oil-degrading bacteria. Previous biostimulation studies using WS reported degradation rates between 59 and 85% during a period of 1–3 months (Vasudevan and Rajaram, 2001; Marin et al., 2006; Rojas-Avelizapa et al., 2007). Other studies have used WS as a source of inorganic nutrients such as N and P and organic nutrients such as cellulose and hemicellulose (Zhang et al., 2008; Llado et al., 2015). It was postulated that the decomposition products of cellulose and hemicellulose promote the growth of bacteria including the oil-degrading types. WS was also used to enhance bioremediation in salty contaminated soils, where high salt levels may inhibit microbial growth, by cutting capillaries and decreasing salt accumulation at the soil surface (Zhang et al., 2008). This effect was shown to result in a remarkable increase in bacterial biomass and in an increase in TPH degradation from 29 to 48% (Zhang et al., 2008).

### Bacterial Diversity and its Changes in the Untreated Desert Soil

MiSeq data showed shifts in the bacterial community structure of untreated as well as in biostimulated soils. The original desert soil was dominated by sequences belonging to the bacterial groups Alphaproteobacteria, Gammaproteobacteria and Firmicutes, and these groups have been previously encountered in oil-contaminated soils, including desert soils (Militon et al., 2010; dos Santos et al., 2011; Sutton et al., 2013). Several members of these bacterial groups are known for their ability to degrade aliphatic and aromatic hydrocarbons (Alonso-Gutierrez et al., 2008; Lafortune et al., 2009; Vila et al., 2010; Kostka et al., 2011). Among the detected genera in our desert soil that potentially contain hydrocarbon-degrading species were *Sphingomonas, Phenylobacterium, Sphingopyxis*, *Pseudomonas, Halomonas*, and *Alcanivorax*. The detection of these genera in the original soil suggests that oil-degradation could be naturally occurring in the soil even without any treatment. This process, which relies on the activity of indigenous oil-degrading microorganisms, is known as natural attenuation and has become an accepted low-risk and cost-effective bioremediation approach (Margesin and Schinner, 2001; Bento et al., 2005; Ruberto et al., 2009). The produced CO_2_ levels and the measured natural degradation by GC–MS analysis in the untreated soil support the occurrence of natural biodegradation in the untreated soil.

Species belonging to the Alphaproteobacterial genera *Sphingomonas*, *Phenylobacterium*, and *Sphingopyxis* in the original soil have been previously detected in oil-contaminated environments and have been known for many years to contain degraders of PAHs (Tao et al., 2007; Lan et al., 2011; Yang et al., 2014; Zhang et al., 2014; Ronca et al., 2015). For instance, *Sphingomonas* sp. was shown to utilize phenanthrene as its exclusive source of carbon and energy (Tao et al., 2007) and to degrade 83% of applied diesel and 3–79% of mixed PAHs (Cui et al., 2008). *Phenylobacterium* and *Sphingopyxis* spp. were also shown to degrade several aromatic hydrocarbons as a carbon source (Eberspaecher and Lingens, 2006; Kertesz and Kawasaki, 2010; Lan et al., 2011; Kim et al., 2014; Yang et al., 2014). Similarly, strains of the genera *Pseudomonas, Alcanivorax* and *Halomonas*, which prevailed the class Gammaproteobacteria, are renowned for their global distribution in contaminated sites and for their ability to degrade hydrocarbons (Hara et al., 2003; Schneiker et al., 2006; Mnif et al., 2009; Linda and Bouziane, 2012; Oyetibo et al., 2013). The two dominant genera of the phylum Firmicutes; *Bacillus* and *Streptococcus*, have also been previously detected in contaminated soils with an active role in the degradation process (Makut and Ishaya, 2010; Mansur et al., 2014).

The incubation of the soil for 64 days in microcosms, even without any treatment, induced clear shifts in the microbial community composition. It is known that confined incubation of bacterial samples results in changes in the composition of bacterial community structure, an effect known as “bottle effect” (Hammes et al., 2010). While the abundance of Flavobacteria increased in S_64_-A, the abundance of Actinobacteria increased in S_64_-A and S_64_-B and the abundance of Firmicutes increased in S_64_-C. Genera affiliated to Firmicutes and Actinobacteria are known for their vast distribution in oil-contaminated soils and they contain strains that were able to degrade hydrocarbons. Actinobacteria play a central role in the decomposition of organic matter and recycling of nutrients (Prince et al., 2010). Among the detected Actinobacterial genera known to degrade hydrocarbons are *Corynebacterium and Micrococcus* (Santhini et al., 2009; Oyetibo et al., 2013; Hassanshahian et al., 2014).

### Post-biostimulation Bacterial Community Shifts

The relative abundance of the bacterial groups exhibited high variability among the triplicate samples of each treatment, making it difficult to attribute specific shifts in the bacterial community to the applied treatment. Nevertheless, NMDS ordination based on the different treatments placed the microbial communities in separate clusters and this dissimilarity was supported by an ANOSIM *R* value of 0.66. This suggests that, in spite of the heterogeneity in the triplicate soil samples of each treatment, the species composition still varied between the different treatments. Moreover, the persistence of the same bacterial groups after treatment indicates that these groups still favored the new conditions. In the SG-treated soils, the relative abundance of Alphaproteobacteria remained the same or increased in all replicates, whereas the abundance of Firmicutes, Gammaproteobacteria and Chloroflexi increased in some replicates but not in others. The alphaproteobacterial genera *Sphingomonas* and *Brevundimonas*, which constituted 35 and 20.4% of the total sequences, respectively are known to include species that degrade petroleum compounds (Ijah and Ukpe, 1992; Baraniecki et al., 2002; Vazquez et al., 2013). The persistence of the Firmicutes group after biostimulation with SG indicates that this group constitutes a stable and vital component of the bacterial community structure of the desert soil. Sequences in this group are prevailed by *Bacillus, Salirhabdus* and *Virgibacillus*, which are known to have oil-degrading species (Kothari et al., 2013; Borah and Yadav, 2014; Roy et al., 2014; Albokari et al., 2015). The detection of sequences belonging to the class Chloroflexi only in the treatment with SG and not in others, strongly suggests that these bacteria have been growing in the sludge and brought into the soil. The green non-sulfur Chloroflexi bacteria were previously found abundant in wastewater treatment plants (Bjornsson et al., 2002; Kragelund et al., 2007; An et al., 2013).

In the soil amended with SB, Firmicutes persisted while Gammaproteobacteria increased in abundance to reach 25–63% of total sequences, of which 97% were affiliated with the genus *Halomonas*. The detection of high abundance of *Halomonas* in this treatment suggests that these bacteria were introduced by the SB or favored the growth on the organics therein. This assumption is supported by the produced CO_2_ of soybean alone, which indicated the presence of an active bacterial community. Although the genus *Halomonas* is known to include oil-degrading species (Mnif et al., 2009; D’Ippolito et al., 2011), most of the detected sequences were affiliated to the species *Halomonas xinjiangensis*, which was isolated from a pristine soil and is unable to degrade hydrocarbons (Guan et al., 2010). The amendment of the soil with WS induced the most remarkable shift in the bacterial community, indicating that WS created different conditions in this treatment that favored the growth of Firmicutes species. Although the phylum Firmicutes includes both aerobic and anaerobic microorganisms, all detected species in this treatment belonged to aerobic *Bacilli*-related species. This suggests that WS probably acted as a bioventing agent and increased oxygen penetration in the soil. Bacilli strains are known to include many oil-degrading species and were even detected in crude oils (Ijah and Ukpe, 1992; Khanna et al., 2012; Borah and Yadav, 2014). An interesting feature of *Bacilli* strains is their ability to produce biosurfactants, which increase the bioavailability of oil through emulsification and consequently facilitate the degradation process (Cubitto et al., 2004; Yamane et al., 2008; Perfumo et al., 2010; Najafi et al., 2011; Chandankere et al., 2013).

We conclude that SG, WS, and SB are suitable bioremediation agents that can be successfully used to enhance the activity of oil-degrading bacteria and facilitate the degradation of petroleum contaminants in desert soils. SG and WS had a stronger stimulatory effect on the soil’s respiration activities and oil degradation than SB. Microbial community analysis revealed the dominance of sequences affiliated to Alphaproteobacteria, Gammaproteobacteria, and Firmicutes in the original soil, although there was a clear heterogeneity among the triplicate samples. The relative abundance of these groups showed variations after the addition of the biostimulating agents, with the most prominent shift in the case of WS-treated soils, where almost the whole community was composed of Bacilli.

## Author Contributions

SA-K and RA designed the experiments, SA-K performed the experiments and the chemical analysis using GC–MS, RA did the molecular work and the bioinformatics analysis. SA-K and RA wrote the manuscript.

## Conflict of Interest Statement

The authors declare that the research was conducted in the absence of any commercial or financial relationships that could be construed as a potential conflict of interest.
